# The Role of Mitochondrial Metabolism, AMPK-SIRT Mediated Pathway, LncRNA and MicroRNA in Osteoarthritis

**DOI:** 10.3390/biomedicines10071477

**Published:** 2022-06-22

**Authors:** Hao-Yu Liu, Chi-Fen Chang, Cheng-Chang Lu, Shun-Cheng Wu, Bin Huang, Tsung-Lin Cheng, Sung-Yen Lin, Cheng-Jung Ho, Mon-Juan Lee, Chung-Da Yang, Ying-Chun Wang, Jhong-You Li, Ping-Cheng Liu, Chun-Wang Wei, Lin Kang, Chung-Hwan Chen

**Affiliations:** 1 Orthopaedic Research Center, College of Medicine, Kaohsiung Medical University, Kaohsiung 80708, Taiwan; artherb02@gmail.com (H.-Y.L.); cclu0880330@gmail.com (C.-C.L.); shunchengwu@hotmail.com (S.-C.W.); junglecc@kmu.edu.tw (T.-L.C.); tony8501031@gmail.com (S.-Y.L.); rick_free@mail2000.com.tw (C.-J.H.); mark163333@gmail.com (J.-Y.L.); liupingcheng@yahoo.com.tw (P.-C.L.); 2 Department of Orthopedics, Kaohsiung Medical University Hospital, Kaohsiung Medical University, Kaohsiung 80708, Taiwan; ycwang.ow@gmail.com; 3 Regeneration Medicine and Cell Therapy Research Center, Kaohsiung Medical University, Kaohsiung 80708, Taiwan; 4 Department of Orthopedics, College of Medicine, Kaohsiung Medical University, Kaohsiung 80708, Taiwan; 5 Department of Anatomy, School of Medicine, China Medical University, Taichung 40402, Taiwan; cfchang@mail.cmu.edu.tw; 6 Department of Orthopedics, Kaohsiung Municipal Siaogang Hospital, Kaohsiung 812, Taiwan; 7 Department of Biomedical Science and Environmental Biology, College of Life Science, Kaohsiung Medical University, Kaohsiung 80708, Taiwan; huangpin2@yahoo.com.tw; 8 Department of Physiology, College of Medicine, Kaohsiung Medical University, Kaohsiung 80701, Taiwan; 9 Graduate Institute of Clinical Medicine, College of Medicine, Kaohsiung Medical University, Kaohsiung 80708, Taiwan; 10 Department of Bioscience Technology, Chang Jung Christian University, Tainan 71101, Taiwan; mjlee@mail.cjcu.edu.tw; 11 Innovative Research Center of Medicine, Chang Jung Christian University, Tainan 71101, Taiwan; 12 Graduate Institute of Animal Vaccine Technology, College of Veterinary Medicine, National Pingtung University of Science and Technology, Pingtung 912301, Taiwan; cdyang@mail.npust.edu.tw; 13 Ph.D. Program in Biomedical Engineering, College of Medicine, Kaohsiung Medical University, Kaohsiung 80708, Taiwan; 14 Department of Orthopedics, Kaohsiung Municipal Ta-Tung Hospital, Kaohsiung 80145, Taiwan; 15 Department of Healthcare Administration and Medical Informatics, Kaohsiung Medical University, Kaohsiung 80708, Taiwan; 16 Department of Obstetrics and Gynecology, National Cheng Kung University Hospital, College of Medicine, National Cheng Kung University, Tainan 701, Taiwan; 17 Institute of Medical Science and Technology, National Sun Yat-Sen University, Kaohsiung 80420, Taiwan; 18 Graduate Institute of Materials Engineering, College of Engineering, National Pingtung University of Science and Technology, Pingtung 912301, Taiwan

**Keywords:** osteoarthritis, mitochondria, AMP-activated protein kinase (AMPK), sirtuins (SIRT), long non-coding RNA (lncRNA), microRNA (miRNA)

## Abstract

Osteoarthritis (OA) is the most common joint disease characterized by degeneration of articular cartilage and causes severe joint pain, physical disability, and impaired quality of life. Recently, it was found that mitochondria not only act as a powerhouse of cells that provide energy for cellular metabolism, but are also involved in crucial pathways responsible for maintaining chondrocyte physiology. Therefore, a growing amount of evidence emphasizes that impairment of mitochondrial function is associated with OA pathogenesis; however, the exact mechanism is not well known. Moreover, the AMP-activated protein kinase (AMPK)–Sirtuin (SIRT) signaling pathway, long non-coding RNA (lncRNA), and microRNA (miRNA) are important for regulating the physiological and pathological processes of chondrocytes, indicating that these may be targets for OA treatment. In this review, we first focus on the importance of mitochondria metabolic dysregulation related to OA. Then, we show recent evidence on the AMPK-SIRT mediated pathway associated with OA pathogenesis and potential treatment options. Finally, we discuss current research into the effects of lncRNA and miRNA on OA progression or inhibition.

## 1. Introduction

Osteoarthritis (OA) is a degenerative and debilitating joint disease associated with the symptoms and signs of joint pain, swelling, stiffness, mobility limitation, and joint deformity [1]. According to data from the United States Centers for Disease Control and Prevention in 2015, OA affected 54.4 million adults in the U.S., and the number is expected to reach 78.4 million by 2040, which indicates the large and growing problems for clinical and public health systems [2,3]. OA is a complicated disease with multifactorial etiologies, including aging, obesity, genetic predisposition, and mechanical overloading [4]. Cartilage degeneration, chondrocyte senescence and apoptosis, degradation of the extracellular matrix, inflammation of the synovium, and subchondral bone dysfunction are the core pathological changes [4,5,6,7]. Clinical symptoms and radiographic changes include chronic pain, joint instability, tenderness, stiffness, joint deformities, thinning of the cartilage, subchondral bone sclerosis, and radiographic joint space narrowing [8]. At present, there are no effective interventions or disease-modifying treatments for osteoarthritis, and pharmacological treatments are confined to symptom relief and function restoration rather than stopping the progression of the disease. The only end-point treatment for advanced-stage OA is joint replacement surgery [9,10]. We still need a better understanding of the molecular mechanisms related to OA pathogenesis in order to design potential molecular targets for prevention and treatment.

Mitochondria are double-membrane organelles that convert organic molecules (e.g., glucose, amino acids, and fatty acids) into adenosine triphosphate (ATP) via the electron transport chain (ETC) and oxidative phosphorylation [11,12]. Hence, mitochondria are traditionally described as a cell’s power factory. In addition, articular chondrocytes have been regarded as highly glycolytic cells in past studies [13,14]. However, recent studies have found that aerobic respiration seems to be an important energy source in articular chondrocytes, accounting for approximately 25% of their ATP production [15,16]. Consequently, mitochondrial aerobic respiration is one of the essential energy-producing pathways in articular chondrocytes. Besides energy production, mitochondria also play important roles in many cellular functions, including cell differentiation, senescence, and cell death. Recent investigations demonstrated that the alteration of mitochondrial function is associated with OA pathogenesis [16,17,18]. In addition, more and more evidence suggests that mitochondrial dysfunction is broadly involved in multiple molecular mechanisms of OA progression, including excess oxidative stress, energetic metabolism change, respiratory chain dysfunction, cell senescence, autophagy and apoptosis, and calcium homeostasis [19,20].

In addition, recent studies have shown that the AMP-activated protein kinase (AMPK) and sirtuin (SIRT) pathways are important for signal transduction to regulate mitochondrial physiological function. They are also related to the normal metabolism of chondrocytes, including extracellular matrix (ECM) production, cytoskeleton rearrangement, autophagy, and apoptosis, which are the core pathogenesis of OA. Furthermore, increasing evidence shows that long non-coding RNA (lncRNA) and microRNA (miRNA) are closely related to it. LncRNA and miRNA regulate the expression levels of downstream genes and transmit the biological information to the target cells. Each has different regulatory effects on ECM degradation, chondrocyte apoptosis and proliferation, inflammatory responses, and autophagy activity, which indicates their great potential as treatment options for OA.

Therefore, in this review, we first discuss how mitochondrial metabolism dysfunction can lead to OA pathogenesis. Then, we unravel the AMPK-SIRT pathway, lncRNA, and microRNA in association with OA pathogenesis, and discuss potential therapeutic strategies.

## 2. Mitochondrial Metabolism Dysfunction to OA Pathogenesis

**Figure 1:**
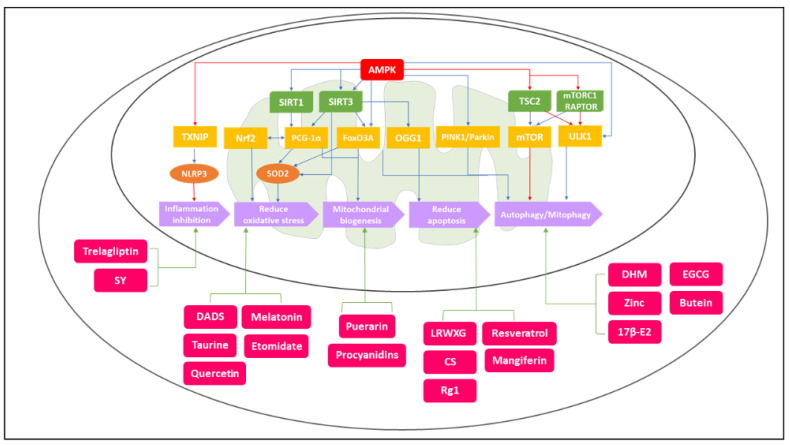
The diagram shows the AMPK–SIRT signaling pathways related to OA pathogenesis and the potential treatment options for OA targeting at mitochondrial pathways. The outer circle is the cell membrane, and the inner circle is the mitochondrial outer membrane. The red, green, yellow, and orange blocks represent the regulatory molecules and the purple blocks represent the final metabolic responses in the AMPK–SIRT signaling pathways. The light-red blocks represent potential treatment drugs. The blue arrow means activation, and the red arrow means inhibition in the signaling pathways. The activation of AMPK–SIRT1/SIRT3–PCG–1α/FoxO3A, AMPK–SIRT3–OGG1, AMPK–PINK1/Parkin, and AMPK-ULK1/mTOR could reduce oxidative stress, promote mitochondrial biogenesis, reduce apoptosis, enhance autophagy/mitophagy, and inhibit inflammatory responses. In addition, the targeting metabolic pathways of potential treatment options—DADS, Taurine, Quercetin, Melatonin, LRWXG, CS, Rg1, DHM, Puerarin, Zinc, 17β-E2, Trelagliptin, Etomidate, SY, EGCG, Resveratrol, Procyanidins, Butein, and Mangiferin—are shown above. The green arrows indicate the metabolic effects of these potential treatment drugs.

Mitochondria are known to be dynamic and complex organelles responsible for numerous physiological processes in chondrocytes related to energy production and cell growth and proliferation. Recent studies have also reported that mitochondrial metabolism dysfunction could affect several pathways, causing the generation of reactive oxygen species (ROS), inflammation regulation, chondrocyte senescence, matrix catabolism, apoptosis induction, and calcium homeostasis [15,21,22,23]. Consequently, alterations in mitochondrial metabolic function are associated with OA pathological processes as mentioned above. Furthermore, recent investigations showed that AMPK-SIRT functions as important biosensors that regulate a variety of physiological processes via targeting downstream regulators, as shown in Figure 1. These downstream regulatory factors then lead to different metabolic responses, including inflammation inhibition, oxidative stress reduction, mitochondrial biogenesis, apoptosis prohibition, autophagy, and mitophagy [24,25,26]. All of these metabolic responses regulated by AMPK-SIRT pathways are closely related to physiological mitochondrial functions. Therefore, the imbalance or disruption of these important signaling pathways may further lead to mitochondrial dysfunction, and eventually, evolve into OA.

In addition to AMPK-SIRT regulation, recent studies have yielded evidence on the interaction between lncRNA and miRNA in relation to OA pathogenesis. LncRNA, which binds to miRNAs to inhibit their combining with downstream genes, is crucial for metabolic reactions and homeostasis of articular chondrocytes [27]. Together with miRNA, it focuses on promoting or inhibiting ECM degradation, chondrocyte apoptosis and proliferation, and inflammation responses, which are all related to OA pathogenesis [28,29,30]. Therefore, LncRNA and miRNA are integral to the metabolic regulation of chondrocytes, and their downstream targets may interconnect with AMPK-SIRT signaling pathways. Hence, their abnormal expression in chondrocytes could cause metabolic dysfunction and lead to pathological progression of OA.

### 2.1. Mitochondrial Respiratory Chain (MRC) in Osteoarthritis

The mitochondrial membrane potential is generated by proton pumps (Complexes I, III, and IV), which are located on the inner membrane and generate transmembrane potential energy in the form of hydrogen ions that create an electrochemical gradient during oxidative phosphorylation (OXPHOS) [31]. Adenosine triphosphate (ATP), which functions as the primary energy source for several physiological reactions in human tissue cells, is mainly generated by the mitochondrial respiratory chain (MRC). However, in chondrocytes, the ATP generation mostly relies on glycolysis because metabolism in chondrocytes occurs under a relatively low oxygen supply, which ranges from 5 to 10% at the superficial zone to <1% in the deep zone [13,32].

However, in OA cartilage, due to degradation (thinning, micro-cracks, and even disappearance), chondrocytes in the deep zone receive more oxygen stimulation. In a relatively high oxygen concentration environment, the dominant chondrocyte metabolic state switches from glycolysis to aerobic respiration, resulting in the production of more ROS byproducts [17,33,34]. Meanwhile, other studies hypothesized that chondrocytes, which have an anti-inflammatory effect on oxidative phosphorylation, turn pro-inflammatory when glycolysis is used for energy production, which leads to the progression of OA [35,36,37]. Other studies also demonstrated that the abnormal glycolytic process may lead to chondrocyte dysfunction via the downregulation of glycolytic enzymes. Qu, J et al. found that restoring glycolysis can reduce the expression of endoplasmic reticulum (ER) stress-associated genes and matrix metalloproteinase-13 (MMP-13), which are induced by inflammatory factors such as tumor necrosis factor-α (TNF-α) or IL-1β. [38,39]. Therefore, the link between impaired glycolysis and OXPHOS dysfunction contributory to OA needs further study.

Currently, the standard evaluation of mitochondrial function is the analysis of respiratory chain enzyme complexes, citrate synthase (CS), and changes in mitochondrial membrane potential (ΔΨm). Recent studies have shown that chondrocytes in OA reduced activities of complexes II and III and impaired ΔΨm compared with normal chondrocytes. These findings suggest that malfunction of the mitochondrial respiratory chain is involved in the pathophysiology of OA chondrocytes [11,40]. López-Armada, M. J. et al. revealed that tumor necrosis factor-α (TNF-α) and interleukin-1 (IL-1) significantly decreased the activity of complex I and the production of ATP, and caused depolarization of the mitochondria, which play a key role in cartilage degradation [41]. Furthermore, Cillero-Pastor et al. reported that mitochondrial respiratory chain (MRC) dysfunction modulates matrix metalloproteinases (MMPs) expression, and leads to ECM degradation of the articular cartilage, which is a crucial component in OA pathogenesis [42]. 

Recent studies have reported that mitochondria are critical mechanotransducers, which connect the extracellular mechanical signals with intracellular signaling pathways [43,44]. Bartell et al. reported that mechanical overloading will induce mitochondrial dysfunction including mitochondrial depolarization and rapid structural changes in mitochondria, within a few minutes after injury, and finally cause cartilage damage [45]. The mechanically induced distortion of mitochondrial membrane polarity, the ETC, and the cristae structure can be related to the physical linkage between the cartilage ECM, cytoskeleton, and mitochondria, resulting in compromised respiratory capacity, release of ROS, and reduced cell viability [16,46,47].

### 2.2. Reactive Oxygen Species (ROS) in Osteoarthritis

ROS are free radicals formed by the normal cellular metabolism of oxygen. The main ROS produced by chondrocytes are nitric oxide (NO), superoxide anion (O^2−^), and their derivative radicals, including peroxynitrite (ONOO-) and hydrogen peroxide (H_2_O_2_) [17,18,23]. Because of unpaired electrons, ROS are short-lived, highly reactive, and unstable. They interact with lipids, proteins, and DNA leading to increased oxidative stress [22]. Recent research revealed two major processes involved in the generation of ROS: the mitochondrial and non-mitochondrial pathways. In the former, mitochondria, which are considered to be a cell’s powerhouses, produce ROS during OXPHOS, which are involved in cartilage degradation and chondrocyte cell death [48,49]. Under normal physiological conditions, ROS act as second messengers for intracellular signaling pathways, regulating gene expression, immune responses, and metabolism homeostasis [18]. However, under pathological conditions, excessive ROS contribute to joint inflammation by inducing cytokine production. They also cause chondrocyte apoptosis, cartilage degradation from reducing matrix synthesis and activation of MMPs, and mtDNA damage [23,50]. Therefore, the ROS produced by mitochondria play an important role in OA pathogenesis. 

On the other hand, the non-mitochondrial pathway mainly refers to nicotinamide adenine dinucleotide phosphate (NADPH) oxidase. Some studies showed that in pathological conditions, including mechanical overloading and inflammatory mediator stimulation, chondrocytes produce abnormal levels of ROS through NADPH oxidase, which exceeds the antioxidant capacities of the cell, leading to structural and functional cartilage damage such as cell death and matrix degradation [51,52,53]. In addition, Grange, L. et al. reported that NADPH oxidase, which responds to interleukin-1β, is significantly involved in ROS production in chondrocytes, leading to MMP production, which mediates the progressive degradation of cartilage [54,55].

Oxidative stress damage may contribute to chronic and persistent mitochondrial dysfunction because overproduction of ROS impairs mitochondrial respiration, which further increases ROS production, thus forming a vicious cycle. In addition, oxidative stress is essential for the development of certain characteristics of OA, including synovial inflammation, cell senescence and apoptosis, impaired autophagy, and ECM degradation [56]. Lepetsos, P. et al. reported that ROS overproduction contributed to decreasing ECM synthesis by inhibiting the OXPHOS process, which impaired mitochondrial ATP formation, and increased ECM degradation by upregulating the expression of genes encoding MMPs, such as MMP-1, MMP-3, and MMP-13 [57]. Reed et al. also demonstrated that oxidative stress resulting from mitochondria could modulate ECM destruction through the upregulation of the MMP level [50]. The contributions of ROS to autophagy, inflammation, cell senescence, and apoptosis will be discussed later.

On the other hand, antioxidant systems balance the production and elimination of ROS. These systems, the first line of defense, consist of enzymatic and non-enzymatic antioxidants, including glutathione peroxidase (GSH-PX), superoxide dismutase (SOD), and catalase (CAT). In particular, superoxide dismutase (SOD) is crucial for catalyzing the dismutation of the superoxide radical (O^2−^) into harmless molecular oxygen (O_2_) and hydrogen peroxide (H_2_O_2_), thereby protecting cells from oxidative damage [58,59]. Ruiz-Romero, C. et al. reported that the expression of superoxide dismutase 2 (SOD2) is significantly downregulated in OA chondrocytes at both the gene and protein levels. This causes a global alteration of the redox balance in the bone, cartilage, and synovial fluid and further increases the accumulation of ROS [60]. Koike, M. et al. also revealed that the intra-articular injection of a permeable antioxidant effectively suppressed mechanical loading-induced mitochondrial superoxide generation and cartilage degeneration in mice [61]. Consequently, the imbalance between ROS production and antioxidant defense leads to intracellular oxidative stress damage, which raises the possibility that the mitochondrial superoxide balance may be a promising target for the treatment of cartilage degeneration.

The contents in Table 1 represent the mechanism and biological functions of traditionally potential drugs in OA. The metabolic effects of these drugs include reduction in ROS, inhibition of ECM degradation, reduced inflammatory responses, and apoptosis inhibition. On the other hand, the contents in Table 2 represent the classification and biological functions of polyphenol, which is a newly emerging potential drug for OA. There are many kinds of polyphenol, and its physiological effects include autophagy activation, apoptosis inhibition, inflammation attenuation, and mitophagy promotion. All of the metabolism responses are key factors in OA pathogenesis, and we discuss these potential drugs in each chapter.

Some research into drugs that target ROS production is shown in Table 1 and Table 2. Hosseinzadeh, A. et al. reported that diallyl disulfide (DADS), which has antioxidant and anti-inflammatory properties, can increase nuclear transcription factor erythroid-2-like factor 2 (Nrf2) nuclear translocation, and the gene expression of antioxidant enzymes, and it reduced the IL-1β-induced elevation of ROS [62]. Kim, Y. S. et al. revealed that taurine increased cell viability by protecting cells from ROS-induced by H_2_O_2_ exposure [63]. In addition, Qiu, L. et al. showed that LRWXG, a flavonol-type flavonoid ubiquitously present in vegetables, attenuated ROS generation and promote glutathione (GSH) and glutathione peroxidase (GSH-PX) expression levels in an OA rat [64]. Furthermore, Lim, H. D. et al. reported that melatonin, an amine hormone produced by the pineal gland, reduces oxidative stress and inflammatory responses by inhibiting H_2_O_2_ cytotoxicity, inducible nitric oxide synthase (iNOS), and cyclooxygenase-2 (COX-2) gene expression in chondrocytes [65]. According to the studies shown above, antioxidants including DADS, taurine, quercetin, and melatonin are potential drugs for the treatment of OA.

**Table 1:** Classifications and biological functions of drugs in osteoarthritis.

Drugs	Mechanism	Function	Ref.
DADS	Increase Nrf2 nuclear translocation and gene expressions of antioxidant enzymes	Reduce the production of ROS	[62]
Taurine	Scavenge excessive ROS	Decrease the H_2_O_2_-induced ROS production	[63]
Quercetin	Promote the expression of GSH and GSH-PX	Attenuate the generation of ROS	[64]
	AMPK/SIRT1 signaling pathway	Inhibit cartilage ECM degradation	
Melatonin	Inhibit H_2_O_2_ cytotoxicity, iNOS, and COX-2 gene expression	Reduce the oxidative stress and inflammatory responses	[65]
LRWXG	Upregulate the expressions of Bcl-2 and downregulate the expressions of caspase-9, caspase-3, and Bax	Inhibit the apoptosis of chondrocytes	[66]
CS	Inhibit the expressions of caspase-3 and caspase-9	Inhibit the apoptosis of chondrocytes	[67]
Rg1	PI3K/Akt/mitochondrial signaling pathway	Inhibit IL-1β-induced chondrocyte apoptosis	[68]
DHM	AMPK/SIRT3/PGC-1α signaling pathway	Inhibit cartilage degeneration	[69]
	Upregulate SIRT3	Promote mitophagy activity	
Puerarin	AMPK/PGC-1α signaling pathway	Increase mitochondrial biogenesis and attenuate mitochondrial dysfunctions	[70]
Zinc	PINK1-dependent mitophagy pathway	Promote mitophagy activity	[71]
17β-E2	AMPK/SIRT1/mTOR signaling pathway	Induce mitophagy upregulation	[72]
Trelagliptin	AMPK/SOX-9	Ameliorate oxidative stress and inflammatory responses	[73]
Etomidate	Upregulate AMPK signaling	Decrease oxidative stress, degradation of ECM, and chondrocyte senescence	[74]
SY	AMPK/SIRT1/NF-κB pathway	Inhibit degradation of cartilage ECM and inflammatory responses	[75]

**Table 2:** Classifications and biological functions of polyphenol in osteoarthritis.

Drugs	Mechanism	Function	Ref.
EGCG	Decrease the expressions of mTOR	Reduce the chondrocyte apoptosis and activate autophagy	[76]
	Decrease expressions of COX-2 and MMP-13	Attenuate the inflammation on cartilage	
Resveratrol	Suppression of IL-1β, ROS, and p53 production	Inhibit IL-1β-induced degradation of mitochondria and chondrocytes apoptosis	[77,78]
	Inhibit mitochondrial membrane depolarization, PGE2 synthesis, and ATP depletion	Reduce IL-1β-induced catabolic metabolism and chondrocytes apoptosis	
	Activate SIRT1 signaling pathway	Inhibit NO-induced apoptosis	
Procyanidins	AMPK/SIRT1/PGC-1α signaling	Promote mitochondrial biogenesis and proteoglycan homeostasis in chondrocytes	[79]
Butein	AMPK/TSC2/ULK1/mTOR signaling pathway	Activate autophagy and inhibit inflammatory responses in OA chondrocytes	[80]
Mangiferin	Activate AMPK signaling pathway	Inhibit apoptosis, ECM degradation and enhance autophagy in OA chondrocytes	[81]

### 2.3. Chondrocyte Senescence and Mitochondrial Autophagy in Osteoarthritis

Recent research showed that cell studies and animal models point to aging-related and stress-induced oxidative stress as important factors related to chondrocyte senescence [82,83,84]. Senescent cells, which are cell-cycle arrested, are actually metabolically active and associated with the overproduction of cytokines (e.g., interleukins 1 and 6), growth factors (e.g., epidermal growth factor, transforming growth factor β), as well as matrix-degrading enzymes such as the MMPs (e.g., MMP-3, MMP-13), collectively known as the senescence-associated secretory phenotype (SASP) [83,85]. Furthermore, apart from the senescent cell itself, SASP can also induce paracrine senescence in neighboring healthy chondrocytes [86]. Jeon et, al. demonstrated that eliminating senescent cells could diminish the development of post-traumatic OA and increase the growth of articular cartilage [87]. In addition, Brian et al. reported that repeated intra-articular injection of senolytic cells, which target anti-apoptotic pathways, mitigate the development of OA [88]. Furthermore, Xu et al. revealed that senescent cells transplanted into the knee could cause clinical characteristics similar to OA, suggesting that targeting senescent cells could be a strategy for treating OA [89]. Overall, the SASP is highly regulated by mitochondria, and dysfunctional mitochondria can induce cellular senescence associated with SASP presentation [90]. The profile of inflammatory and catabolic mediators in senescent cells contributes to OA pathogenesis and may be the potential target for OA treatment [91].

Autophagy is a cellular process regarded as a mechanism for cell survival when cells become stressed, degrading dysfunctional proteins and macromolecules, and then recycling them to produce materials for protein synthesis [91,92]. Increasing evidence shows that autophagy’s role in OA can be modulated by mitochondria [93,94,95,96]. Some studies showed that limited autophagy is a major characteristic of OA chondrocytes, which cause excessive cellular apoptosis and cartilage degradation, thereby implying that autophagy and apoptosis are mutually regulated [94,97,98]. In addition, Zhong et al. revealed that defects in the activation of mitophagy, a specific form of autophagy that selectively eliminates depolarized and dysfunctional mitochondria, cause pronounced accumulation of damaged mitochondria and excessive IL-1β-dependent inflammation in macrophages [99]. Furthermore, Figueroa et al. reported that MRC complex V inhibition will attenuate autophagy, which protects against mitochondrial dysfunction and enhances chondrocyte apoptosis with increased ROS production and decreased ΔΨm. This suggests that enhancing autophagy may have a chondroprotective effect on OA cartilage degradation [100]. Regarding treatment, Huang, H. T. et, al. showed that (−)-Epigallocatechin 3-gallate (EGCG), the most plentiful bioactive polyphenol of green tea with anti-arthritic, anti-inflammatory, and antioxidant effects, could reduce chondrocyte apoptosis by activating autophagy through a reduction in the expression of the mammalian target of rapamycin (mTOR) and attenuation of cartilage inflammation through decreased expression of COX-2 and MMP-13 [76]. These findings suggested that EGCG may be a disease-modifying drug for preventing OA progression.

### 2.4. Chondrocyte Apoptosis and Calcium Homeostasis Regulation in Osteoarthritis

Apoptosis is the most common form of cell death. It is characterized by a series of morphological and biochemical changes that lead to the formation of apoptotic bodies that are phagocytosed by macrophages [96]. It is well known that apoptosis can be initiated by a variety of microenvironmental perturbations in which mitochondria play a central role (intrinsic apoptosis), or by specific ligand binding to death receptors, including the Fas cell surface death receptor (FAS) and the tumor necrosis factor (TNF) receptor (extrinsic apoptosis) [96,101]. The critical step for intrinsic apoptosis is the irreversible mitochondrial outer membrane permeabilization (MOMP), which leads to the release of cytochrome c and promotes the formation of the supramolecular complex known as apoptosome with apoptotic peptidase activating factor 1 (APAF1), which is responsible for caspase-9 activation. Concerning extrinsic apoptosis, the binding of a specific ligand to death receptors induces a conformational change at their intracellular tails and recruits several proteins, such as the FAS-associated death domain (FADD), and further activates caspase-8, which leads to the proteolytical activation of downstream effector caspases [96,101]. 

There are a large number of apoptotic chondrocytes in OA cartilage, and mitochondrial-induced chondrocytes apoptosis is mediated by the reduction in ΔΨm, overexpression of ROS, mechanical stress, aging, and the inflammatory factors [102]. Maneiro, E. reported that the collapse of ΔΨm is associated with mitochondrial swelling, disruption of the outer mitochondrial membrane, and the release of pro-apoptotic factors from the intermembrane space: cytochrome c, and pro-caspases such as caspase-9, which might lead to a series of events culminating in apoptosis [11,102]. Furthermore, they also revealed that sodium nitroprusside (SNP), an NO donor and a pro-apoptotic stimulus, induces depolarization of the mitochondrial membrane along with reduced activity of MRC complex IV, which eventually causes chondrocyte apoptosis [103]. The mechanical stress on the induction of chondrocyte apoptosis is also involved in mitochondrial-induced chondrocytes apoptosis and is highly connected with calcium homeostasis regulation, which is discussed in the next paragraph [104,105].

The calcium ion (Ca^2+^), one of the most important intracellular second messengers, is responsible for biological functions, such as cell adhesion, metabolism, secretion, proliferation, and apoptosis [106]. Particularly, the calcium stored in mitochondria helps maintain intracellular Ca^2+^ homeostasis [107]. Studies reported that when MRC was directly suppressed, the matrix-vesicle-mediated mineralization of chondrocytes was promoted, and these are present in OA cartilage [15,108,109]. In addition, mechanical stimulation significantly promoted increased cytosolic Ca^2+^ concentration, which mainly relies on two mechanisms: Ca^2+^ influx from the extracellular environment and Ca^2+^ release from intracellular stores such as the endoplasmic reticulum (ER) [106,110]. Furthermore, research revealed that calcium is released from the endoplasmic reticulum via the ryanodine receptor and is taken up by the mitochondria via the uniport transporter following a single-impact load. This causes mitochondrial depolarization and caspase 9 activation, which eventually leads to chondrocyte apoptosis and OA progression [111]. 

Under physiological cell stress, mitochondria can take up Ca^2+^ from cytosolic Ca^2+^ or the ER, and store it transiently to maintain homeostasis and regulate mitochondrial function [105,112]. Wann et al. reported that under physiological stress, elevated intracellular Ca^2+^ is beneficial for matrix synthesis [113]. However, with supra-physiological elevations of intracellular Ca^2+^, mitochondrial Ca^2+^ uptake can increase 10- to 1000-fold and begin to shape Ca^2+^ dynamics [105]. Huser and Davies showed that impact-induced chondrocyte apoptosis and mitochondrial depolarization were significantly decreased when the amount of calcium available to chondrocytes was reduced, suggesting that calcium homeostasis appeared to be involved in articular chondrocyte apoptosis following excessive mechanical stimulation in the single-impact-loading model [111]. Moreover, the accumulation of excessive calcium in the mitochondrial matrix leads to the formation of the permeability transition pore (PTP), which in turn causes the collapse of the mitochondrial transmembrane potential. The result is the release of Ca^2+^ pro-caspases such as caspase-9, and mitochondrial proteins such as cytochromes, as well as mitochondrial swelling, and cell apoptosis, as the abovementioned intrinsic pathway [114,115]. 

Several studies are concerned with drugs that focus on the mitochondrial apoptotic pathway, and these are listed in Table 1 and Table 2. Shao, X. et al. showed that a lower range of the molecular weight of xanthan gum (LRWXG) could inhibit the apoptosis of chondrocytes by upregulating the expression of B-cell lymphoma 2 (bcl-2) and downregulating the expression of active caspase-9 and caspase-3, and Bcl-2-associated X protein (bax) [66]. Liu, Q. et al. reported that chondroitin sulfate (CS), an important component of cartilage ECM, could increase cell survival and decrease chondrocyte apoptosis by inhibiting expression of caspase-3 and caspase-9 [67]. Huang, Y. et al. revealed that ginsenoside Rg1 (Rg1) could protect chondrocytes from IL-1β-induced apoptosis via targeting the phosphatidylinositol 3-kinase (PI3K)/protein kinase B (PKB) signaling pathway by inhibiting caspase-3 activation [68]. Csaki, C. et al. indicated that resveratrol, a natural polyphenolic compound with anti-inflammatory and antioxidative properties, inhibited IL-1β-induced degradation of mitochondria and apoptosis in chondrocytes via suppression of IL-1β, ROS, and production of tumor suppressor protein p53 [77]. Dave, M. et al. also reported that resveratrol could protect against IL-1β-induced catabolic effects and prevent chondrocyte apoptosis by inhibiting mitochondrial membrane depolarization, COX-2 derived PGE2 synthesis, and ATP depletion [78]. Based on these results, we concluded that anti-apoptosis drugs may be potential treatments for treating OA.

### 2.5. mtDNA Haplogroups in Osteoarthritis

Mitochondria, which is well known as “maternal inheritance”, have their own genomic DNA [116]. Mitochondrial DNA (mtDNA) encodes 13 polypeptide subunits essential for the OXPHOS process, 2 ribosomal RNAs (rRNAs), and 22 transfer RNAs (tRNAs) in human cells [117,118]. The mitochondrial genome, maternally inherited, has very high rates of mutation and sequence evolution; therefore, after the sequential accumulation of mtDNA mutations, they further establish stable polymorphic sites in various regions of the mtDNA, which define mitochondrial haplogroups [119,120]. Blanco et al. reported that mtDNA, which persists with high-frequency, continent-specific polymorphisms, enables humans to adapt to different climates and influences health [121]. In addition, studies revealed that mtDNA haplogroups are associated with the prevalence, incidence, and progression of OA in various populations, and with comorbidities that are closely related to various OA phenotypes [19,121]. 

In recent studies, European mtDNA haplogroup J was notably correlated with a decreased risk of knee OA in Spain [122,123], and individuals from the United Kingdom (UK) carrying the haplogroup T showed a decreased risk of knee OA [124]. In addition, a recent meta-analysis, which included 3217 subjects, showed that the mtDNA haplogroup J was associated with a lower incidence of knee OA over an 8-year period [125]. Furthermore, in a study from southern China, mtDNA haplogroup G exhibited an increased risk of OA occurrence, and mtDNA haplogroup B seemed to be a protective factor for OA occurrence [126]. Moreover, mtDNA haplogroup cluster TJ showed a lower proportion in OA cases, and both mtDNA haplogroup cluster TJ and haplogroup T revealed a reduced risk of radiographic knee OA progression [127,128]. Finally, some research has reported that patients carrying mtDNA haplogroup H are apt to require total joint replacement surgery and have a greater risk of radiographic OA progression compared with non-H patients [129]. 

There are different viewpoints to explain the associations between mtDNA haplogroups and OA. First, the associations could reside in the subtle functional differences between haplogroups, which eventually influence ROS formation, ATP production, heat generation, and apoptosis in the mitochondria [130]. Second, some studies reported that the protective role of these haplogroups may be attributed to a lower rate of aerobic respiration, decreased production of inflammatory cytokines, and reduced expression of a mitochondrially related pro-apoptotic gene [125,131]. Fernández-Moreno et al. observed that haplogroup J is an uncoupled haplogroup, which presents a protective role against ROS production and inflammatory response; on the other hand, haplogroup H is more efficiently coupled and results in more ROS generation [125]. Martínez-Redondo et al. also showed that haplogroup H presented a higher maximal oxygen uptake than haplogroup J, and had a positive correlation with greater ROS production and mitochondrial oxidative damage [132]. As not all patients carrying the same haplogroups respond in the same way and there are still many parameters that can influence the biological function of mitochondrial haplogroups, precise identification of molecular mechanisms between each haplogroup and OA pathogenesis need further research [20,133].

## 3. AMPK and SIRT Pathway in Related to OA Pathogenesis

AMP-activated protein kinase (AMPK) and sirtuins (SIRT) are important biosensors that are activated in response to high AMP/ATP and ADP/ATP ratios [134]. AMPK activation restores ATP levels by inhibiting ATP-consuming biosynthetic pathways while simultaneously activating pathways that regenerate ATP through the breakdown of macromolecules, including upregulating glucose uptake, fatty acid oxidation, and glycolysis [135,136]. Some studies have reported that AMPK regulates energy metabolism through activating downstream SIRT activity and inhibiting mTOR-activated pathways, which further enhances oxidative metabolism, mitochondrial biogenesis, and lifespan prolongation [134,137,138]. In addition, recent studies have also revealed that one significant function of AMPK is to promote mitochondrial health, and multiple newly discovered targets of AMPK are involved in various aspects of mitochondrial homeostasis, including autophagy and mitophagy, which are involved in OA pathogenesis [139]. 

Sirtuins, which are a family of highly conserved protein modifying enzymes, detect fluctuations in the NAD^+^/NADH ratio and regulate mitochondrial physiology and cell metabolism [140]. There are seven mammalian sirtuins, SIRT1-7, which function to regulate cell metabolism and modulate stress responses in many tissues [141]. Among these sirtuins, the SIRT3, SIRT4, and SIRT5 are localized in mitochondria, and regulate basic mitochondrial biology, including energy production, metabolism, apoptosis, and intracellular signaling [141,142,143].

As mentioned above, AMPK and SIRT together regulate a variety of cellular physical activities. Here, we summarize the mechanism of action of AMPK and SIRT on ECM production, mitochondrial biogenesis, autophagy, mitophagy, oxidative stress, and inflammation inhibition, which is shown in Figure 1. A thorough understanding of the functions and downstream targets of AMPK and SIRT are necessary not only to understand their part in cellular metabolism but also to search for therapeutic intervention in many diseases, including OA.

### 3.1. AMPK and SIRT Regulate ECM (Extracellular Matrix) Production and Mitochondrial Biogenesis in Chondrocytes

AMPK activation can increase ATP generation, which is beneficial for synthesizing ECM components. Some studies reported that physiological dynamic compression loading alters central metabolism in chondrocytes, in which AMPK has an important role in promoting the production of amino acid precursors and glycosaminoglycan (GAG) for ECM synthesis [144,145]. In addition, the previous study showed that upregulation of SIRT1 expression may inhibit OA chondrocyte apoptosis and ECM degradation by increasing Bcl-2 expression and decreasing Bax, MMP1, and MMP13 expression [146].

AMPK and SIRT regulate mitochondrial biosynthesis in chondrocytes via the AMPK-SIRT1/SIRT3-PCG-1α (peroxisome proliferator-activated receptor-γ coactivator 1 alpha) signaling pathway [147,148]. Wang et al. reported that the activated AMPK–SIRT1–PGC-1α pathway increases mitochondrial biogenesis and reverses pro-catabolic responses in chondrocytes, consequently limiting OA progression [148]. Other studies also showed that the impaired activation of AMPK is a key factor in OA chondrocytes and that activated AMPK upregulates the expression of PGC-1α and Forkhead box O3A (FoxO3A) in human chondrocytes, which promotes mitochondrial biogenesis and decreases oxidative stress [70,140]. AMPK and SIRT regulate the activity of the downstream target, such as PGC1α, by phosphorylation and deacetylation [137,150].

PGC-1α is a coactivator with nuclear respiratory factors (NRFs) in transcription and regulates the mitochondrial transcription factor A (TFAM), which is responsible for the mtDNA duplication, mitochondrial biogenesis, and mtDNA stability; moreover, it regulates transcription factor B (TFB), which enhances mtDNA transcription and strengthens mitochondrial biogenesis [151,152]. Nuclear transcription factor erythroid-2-like factor 2 (Nrf2) also has a chondroprotective function in OA, which suppresses MMP expression induced by IL-1β [153]. Nrf2 is a redox-sensitive transcription factors that positively regulates the expression of antioxidants, including Heme oxygenase 1 (HO-1), SOD, GSH-PX, and CAT [62,154]. Additionally, Nrf2 regulates the antioxidant response element (ARE) signal transduction, one of the crucial antioxidant systems that maintain the redox state, and has been regarded as a strategy for eliminating excessive ROS production [155,156]. As a result, PGC-1α and Nrf2 are highly important for mitochondrial biogenesis and reduction in ROS. A study further indicated that PGC-1α activity decreased in OA chondrocytes, which may be a potential target for OA treatment [149]. 

Some studies that focus on drugs targeting AMPK–SIRT pathways related to ECM production and mitochondrial biogenesis are shown in Table 1 and Table 2. Dihydromyricetin (DHM), which has anti-inflammation and antioxidation properties, protected rat chondrocytes from TNF-α-induced cartilage degeneration and maintained mitochondrial homeostasis via the AMPK/SIRT3/PGC-1α signaling pathway [69]. In addition, quercetin suppressed the IL-1β-induced accumulation of nitric oxide (NO), MMP-3, and MMP-13, and maintained the integrity of cartilage ECM by targeting the AMPK–SIRT1 signaling pathway [64]. Moreover, Wang, L. reported that puerarin, which has several biological functions—antioxidation, anti-inflammation, and anti-apoptosis—increased mitochondrial biogenesis, attenuated mitochondrial dysfunctions, and alleviated cartilage damage in OA rats by upregulating AMPK–PGC-1α signaling pathways [157]. Furthermore, Masuda, I. et al. revealed that procyanidins, a class of polyphenols found in apples that has radical scavenging activity, could promote mitochondrial biogenesis and proteoglycan homeostasis in chondrocytes by targeting AMPK/SIRT1/PGC-1α signaling, suggesting that apple polyphenols might be potential drugs for treating OA [79].

### 3.2. AMPK and SIRT Regulate Autophagy and Mitophagy in Chondrocytes

Autophagy is a conserved intracellular process that delivers cytoplasmic elements (e.g., proteins, organelles, and pathogens) to lysosomes for degradation via double-membrane autophagosomes [158]. On the other hand, mitophagy is a specific form of autophagy that selectively eliminates dysfunctional and depolarized mitochondria, which is a core mechanism for controlling mitochondrial quality and quantity [159]. 

Some studies reported that AMPK activates autophagy and mitophagy to remove damaged or defective mitochondria with impaired OXPHOS, whereas mTORC1, the mechanistic target of rapamycin complex 1, blocks this protective process [130,135]. In addition, AMPK triggers the initiation of autophagy by directly phosphorylating and activating ULK1 (unc-51-like autophagy-activating kinase 1); on the other hand, AMPK inhibits mTOR by means of directly phosphorylating the mTOR upstream regulator, which strongly suppresses autophagy by directly phosphorylating and inhibiting ULK1. Studies have reported that AMPK directly phosphorylates the mTOR upstream regulator including TSC2 and the mTORC1 subunit RAPTOR, which results in reducing mTOR activity under conditions of energy stress and inhibiting the phosphorylation on ULK1 that activates autophagy [160,161]. Moreover, Egan, D. et al. reported that cells expressing ULK1 mutants appear to accumulate defective mitochondria, suggesting that the AMPK–ULK1 axis is important not only for general autophagy, but also specifically for the selective removal of damaged mitochondria [162]. Consequently, AMPK promotes autophagy not only by directly activating ULK1, but also by negatively regulating mTORC1. Therefore, the direct links between AMPK and ULK1 are vital for autophagy and mitophagy, which are core events in OA pathogenesis [135,139,163]. 

AMPK also directly regulates downstream mitophagy in chondrocytes via the SIRT1/SIRT3-FoxO3A-PINK1/Parkin signaling pathway, which is involved in clearing damaged mitochondria, limiting ROS production, and improving OA chondrocyte survival under pathological conditions [69,164,165]. Research has shown that AMPK directly phosphorylates FOXO3, which regulates the genes involved in autophagy, and stimulates its transcriptional activity under conditions of energy stress [166,167]. On the contrary, FOXK1 and FOXK2, which are members of the FOX transcription factor family, are phosphorylated by mTOR under a high nutrient environment and compete with FOXO3 to repress genes involved in autophagy [168]. Another important pathway controlling mitophagy is the PINK1–Parkin-mediated removal of depolarized mitochondria. Parkin, an E3 ubiquitin ligase located on the mitochondrial outer membrane, operates in conjunction with PTEN-induced kinase 1 (PINK1), and the phosphorylation of Parkin by PINK1 transforms it into an active E3 ligase that eliminates damaged mitochondria in response to the loss of ΔΨm [169,170]. Ansari, M. Y. et al. reported that the PINK1–Parkin-mediated removal of damaged mitochondria limited ROS generation and further prevented the induction of oxidative stress in OA chondrocytes [171]. Martini, H. also reported that the PINK1-Parkin pathway was activated when mitochondria were damaged or dysfunctional. Then, E3 ubiquitin ligase Parkin from the cytosol further ubiquitinated numerous proteins on the outer mitochondrial membrane, which mediated autophagosome formation and the lysosomal elimination of dysfunctional mitochondria.

Recent research has also reported that autophagy declines with age, which further leads to chondrocyte dysfunction and OA progression. Some studies have indicated that basal autophagy decreases with age, contributing to the accumulation of protein aggregates and ultimately an aging-related articular cartilage dysregulation disease such as OA [100,172]. In addition, the activation of autophagy has chondroprotective effects, indicating that mitophagy is essential for preventing oxidative stress by eliminating damaged mitochondria. This will be discussed later [164,172,173].

There are some potential drugs that focus on the AMPK–SIRT pathways related to mitophagy in OA chondrocytes, and these are shown in Table 1 and Table 2. Huang, L. W. reported that zinc treatment ameliorates the negative effects on energy metabolism and upregulates the PINK1-dependent mitophagy pathway [71]. In addition, Wang, J. indicated that DHM promotes mitophagy by upregulating SIRT3 in rat chondrocytes. This suggests that SIRT3 might be a potential therapeutic target against chondrocyte degeneration and OA [69]. Moreover, Mei, R. et al. showed that 17β-estradiol (17β-E2), which is associated with articular cartilage metabolism and postmenopausal OA, could induce mitophagy upregulation to protect chondrocytes via targeting the AMPK–SIRT1–mTOR signaling pathway [72]. Ansari, M. Y. et al. further indicated that butein, a polyphenol produced by several plants with anti-inflammatory activity, could activate autophagy, blocking the IL-1β-induced expression of IL-6 in OA chondrocytes, by targeting the AMPK/TSC2/ULK1/mTOR pathway [80]. Additionally, Li, Y. et al. revealed that mangiferin, a natural polyphenol with anti-inflammatory and antioxidative effects, protects chondrocytes from apoptosis and ECM degradation and also enhances autophagy in rat OA chondrocytes by activating the AMPK signaling pathway. This suggests that mangiferin might serve as an OA treatment option [81].

### 3.3. AMPK and SIRT Regulate Oxidative Stress and Inflammation Inhibition in Chondrocytes

There is increasing evidence suggesting that AMPK and SIRT may be redox-sensing proteins in the management of ROS levels [135,174]. Some studies have indicated that AMPK and SIRT regulate chondrocyte catabolism and reduce mitochondrial ROS levels via the AMPK–SIRT3–PGC-1α/FoxO3A signaling pathway, and then prevent chondrocyte senescence and death that may inhibit the progression of cartilage damage in OA [149,175]. Olmos, Y. et al. reported that both PGC-1α and FoxO3A reduced cellular oxidative stress by upregulating antioxidant enzymes, including SOD2 (superoxide dismutase 2) and catalase [176]. Kincaid, B et al. revealed that the activation of SIRT3 by AMPK directly activates SOD2 via deacetylation to scavenge ROS, which maintains the intracellular redox balance [177]. In addition, recent research has indicated that SIRT3 contributes to the regeneration of reduced GSH, the major antioxidant responsible for preventing ROS damage, via the deacetylation of isocitrate dehydrogenase 2 (IDH2), a critical component of the mitochondrial antioxidant pathway because of its ability to generate NADPH to regenerate GSH [178,179]. Furthermore, studies have reported that SIRT3 plays a critical role in repairing mtDNA damage, protecting mitochondrial integrity, and preventing cell apoptosis under oxidative stress. It does so by upregulating the acetylation of 8-oxoguanine-DNA glycosylase 1 (OGG1), which is a newly identified target protein that functions in DNA repair [174,175,180].

Apart from regulating oxidative stress, some studies also showed that AMPK and SIRT inhibit inflammation [70,140]. Salminen, A. and Kaarniranta, K. reported that inhibition of NF-κB signaling by AMPK suppresses inflammatory responses, which is significant because inflammation inhibition and autophagic clearance decline with age [181]. Wei, H. et al. also reported that AMPK phosphorylation contributes to the degeneration of TXNIP, which promotes the activation of the NLRP3 inflammasome in cardiomyocytes [182]. In addition, autophagy, which is a negative regulator of NLRP3 inflammasome activation, can be upregulated by AMPK and confer anti-inflammatory effects by improving mitochondrial quality control and removing molecules such as mitochondrial ROS and cytosolic mtDNA, which may be a potential OA treatment target [183]. 

Several research works discussed the drugs that focus on the AMPK–SIRT pathways related to oxidative stress regulation and inflammation inhibition in OA chondrocytes, as shown in Table 1 and Table 2. Liu J. et al. demonstrated that trelagliptin, a selective inhibitor of dipeptidyl peptidase 4 (DPP-4) used for the treatment of type 2 diabetes mellitus (T2DM), ameliorated IL-1β-induced oxidative stress and mitigated IL-1β-induced inflammatory responses via the AMPK/SOX-9 pathway [73]. Furthermore, Yin, M. and Xu, Y. reported that etomidate, an intravenous anesthetic with antioxidant and anti-inflammatory effects, protected chondrocytes from IL-1β-induced oxidative stress, degradation of ECM, and chondrocyte senescence by upregulating AMPK signaling [74]. Moreover, Wang, C. et al. showed that safflower yellow (SY), the main active ingredient in safflowers, which has antioxidant and anti-arthritic properties, prevented degradation of cartilage ECM and inhibited inflammatory responses by regulating the AMPK/SIRT1/NF-κB pathways [75]. Furthermore, Takayama, K. et al. indicated that resveratrol reduced the amount of Bax and increased the amount of Bcl-2 in the mitochondrial fraction, and activated SIRT1 to inhibit NO-induced apoptosis. This indicated that resveratrol and the SIRT1 pathway might contribute to ending the pathogenesis of OA [184].

## 4. Long Non-Coding RNA (lncRNA) and MicroRNA (miRNA) in Related to OA Pathogenesis

The human genome is estimated to consist of around 2% protein-coding RNA (pcRNA); however, the majority of the genome consists of non-coding RNA (ncRNA) [185]. The most common are tRNA, and rRNA, which are essential for protein synthesis. Other ncRNAs, including long and small ncRNAs, regulate gene expression through epigenetic pathways and function as signaling molecules to regulate crucial cellular and metabolic processes [186,187]. In recent years, with the development of molecular biology technology, researchers have gradually discovered the important roles of long non-coding RNA (lncRNA) and MicroRNA (miRNA). Both lncRNA and miRNA are involved in important pathways related to OA pathogenesis, and they can be used as therapeutic biomarkers for evaluating the progression and prognosis of OA.

### 4.1. Relationship between lncRNA and OA Pathogenesis

LncRNA is an ncRNA that transcribes more than 200 nucleotides in length and modulates mRNA stability [188]. Previous studies have reported that, compared with healthy cartilage tissue, the expression levels of different lncRNA—homeobox transcript antisense RNA (HOTAIR), metastasis-associated lung adenocarcinoma transcript 1 (MALAT1), growth arrest-specific transcript 5 (GAS5), PMS1 homolog 2 mismatch repair system component pseudogene 2 (PMS2P2), RP11 445H22.4, H19, CTD 2574D22.4, maternal expression gene 3 (MEG3), and FOXD2-adjacent opposite strand RNA 1 (FOXD2-AS1)—may be upregulated or downregulated in OA cartilage, suggesting that various lncRNAs may modulate the pathological progression of OA. The specific mechanisms are shown in Table 3 [188,189].

Previous studies have shown that HOTAIR, which serves important for cancer progression, is expressed in normal human cartilage [190,191]. Research has also reported that HOTAIR and alpha-1, 2 fucosyltransferase 2 (FUT2) are upregulated in OA cartilage compared with healthy cartilage, resulting in aggravated ECM degradation, chondrocyte apoptosis, and OA progression through the Wnt/β-catenin pathway [192,193]. In addition, Dou, P. et al. reported that HOTAIR strongly promotes the expression of a disintegrin and metalloproteinase with thrombospondin motifs 5 (ADAMTS-5) in human OA articular chondrocytes, which aggravates ECM degradation and promotes OA [194]. Jiang, M. et al. found that the expression of lncRNA PACER is low in OA chondrocytes and decreases cell apoptosis. The inverse correlation between the expression of lncRNA HOTAIR and lncRNA PACER suggested that there was a mutual regulation mechanism between lncRNAs [195]. 

MALAT1 is expressed in many tissues and participates in numerous biological processes [196]. Zhang, Y. et al. reported that MALAT1 levels in OA cartilage were upregulated with AKT3 compared with those in healthy cartilage, whereas the levels of miR-150-5p were downregulated, suggesting that overexpression of MALAT1 inhibited the expression of miR-150-5p and promoted that of AKT3 [197]. In addition, the study showed that overexpression of MALAT1 reduced the expression of MMP13 and ADAMTS 5, promoted the expression of type II collagen in chondrocytes, and inhibited apoptosis and ECM degradation. Consequently, these suggested that lncRNA MALAT1 plays an important role in OA and may participate in OA pathogenesis via the miR-150-5p/AKT3 axis [197]. Studies also reported that Knockdown of MALAT1 in OA synovial fibroblasts increased the expressions of interleukin-8 (IL-8), and also increased the expressions of genes that control cell growth, cell proliferation, and the inflammatory response [198].

Regarding other lncRNAs involved in OA pathogenesis, Song, J. et al. reported that upregulated GAS5 levels in OA chondrocytes negatively regulated miR-21, which increased the expression of MMPs, stimulated apoptosis, and inhibited autophagy [199]. In addition, research showed that H19 lncRNA, which is upregulated in OA chondrocytes, induced chondrocyte damage by promoting the expression of miR-675, and enhanced LPS-induced C28/I2 cell injury by inhibiting miR-130a [200,201]. Previous studies have also reported that lncRNA MEG3 inhibited ECM degradation in cartilage and delayed the progression of OA by modulating the miR-93–TGFBR2 axis. It also attenuated LPS-induced inflammatory damage by targeting the miR-203 that mediates the Sirt1/PI3K/AKT pathways [202,203,204]. Furthermore, previous research demonstrated that FOXD2-AS1, levels of which are upregulated in OA compared with those in healthy cartilage, induced ECM degradation, inflammation response, and promoted OA progression by targeting miR-27a 3p, which upregulated toll-like receptor 4 (TLR4) expression levels [205,206]. In addition, Cao et al. reported that lncRNA FOXD2-AS1 promoted the survival and development of chondrocytes by inhibiting expression of miR-206 and upregulating expression of Cyclin D1 (CCND1), indicating that FOXD2-AS1/miR-206/CCND1 are important for OA pathogenesis [207].

**Table 3:** Classifications and biological functions of lncRNAs in osteoarthritis.

lncRNA	Target/Signaling Pathway	Function	Ref.
HOTAIR	FUT2/Wnt/β-catenin	Increase ECM degradation, chondrocyte apoptosis	[192,193]
	ADAMTS-5	Increase ECM degradation, promoting OA progression	[194]
MALAT1	miR-150-5p/AKT3	Inhibit apoptosis and ECM degradation, promote chondrocyte proliferation	[197]
	IL-8	Promote chondrocyte proliferation, inhibit inflammatory responses	[198]
GAS5	miR-21/MMPs	Stimulate apoptosis, inhibit autophagy	[199]
H19 lncRNA	miR-675	Increase inflammatory responses, aggravate chondrocyte injury	[200,201]
	miR-130a/PTEN/PI3K/Akt		
MEG3	miR-93/TGFBR2	Inhibit ECM degradation	[202,203]
	miR-203/Sirt1/PI3K/AKT	Attenuate inflammatory damage	[204]
FOXD2-AS1	miR-27a-3p/TLR4	Increase ECM degradation and inflammation responses	[205,206]
	miR-206/CCND1	Promote chondrocyte proliferation	[207]

### 4.2. Relationship between miRNA and OA Pathogenesis

In the pathogenesis of OA, miRNA has certain biological functions that mediate ECM metabolism, chondrocyte proliferation and apoptosis, and inflammatory responses. These are shown in Table 4 [208,209]. Recent studies demonstrated that an abundance of miRNAs is involved in ECM metabolism. Wang et al. found that miR-483-5p promotes chondrocyte hypertrophy, ECM degradation, and accelerated the process of OA by directly targeting matrilin 3 (Matn3) and the tissue inhibitor of metalloproteinase 2 (Timp2) [210]. Hu, G. et al. reported that miR-145 alleviated the TNF-α triggered cartilage degradation by inhibiting the phosphorylation of mitogen-activated protein kinase kinase 4 (MKK4) [211]. Li, L. et al. found that the expression of miR-16-5p, which is significantly higher in OA cartilage than in healthy cartilages, reduced expression of type II collagen and aggrecan while inducing expression of MMPs and ADAMTS via the Smad3 pathway [212]. Zhang, Y. revealed that overexpression of miR-21 attenuated the process of chondrogenesis by directly targeting growth differentiation factor 5 (GDF-5) [213].

Chondrocyte proliferation and apoptosis play a key role in OA pathogenesis, and many miRNAs regulate these important pathways. Research has demonstrated that miR-146a targets Smad4 to promote chondrocyte apoptosis, and miR-146b upregulated the activities of proteolytic enzymes, promoted cell apoptosis, and accelerated the development of OA by inhibiting the expression of alpha-2-macroglobulin (A2M) [214,215]. Zhao et al. reported that miR-181a accelerated the apoptosis of chondrocytes by inhibiting the expression of Glycerol-3-Phosphate Dehydrogenase 1-Like Protein (GPD1L) [216]. Li, Z. et al. showed that miR-210 increased chondrocyte proliferation and prompted ECM deposition by inhibiting the expression of hypoxia-inducible factor (HIF)-3α [217]. 

Regarding the inflammatory response, previous studies revealed that several miRNAs are involved in inflammation regulation. Jones, S. revealed that miR-146, which is downregulated in OA cartilage, reduced interleukin-1 beta (IL-1β)-induced TNF-α production [218]. In addition, Bazzonia F. et al. reported that TLR4-activated NF-κB rapidly increased the expression of miR-9, which functions as a negative feedback control of the NF-κB-dependent responses [219]. Furthermore, Park, S. et al. showed that miR-558, which was significantly lower in OA cartilage compared with normal human articular cartilage, controlled cartilage homeostasis by directly targeting COX-2, a major prostaglandin E2 (PGE2) synthetic enzyme involved in chronic inflammation, and the regulation of IL-1β-stimulated catabolic effects in human chondrocytes [220].

**Table 4:** Classifications and biological functions of mi-RNAs in osteoarthritis.

mi-RNA	Target/Signaling Pathway	Function	Ref.
miR-483-5p	Matn3	Promote ECM degradation and chondrocyte hypertrophy	[210]
	TIMP2		
miR-145	MKK4	Alleviate cartilage degradation	[211]
miR-16-5p	Smad3	Increase ECM degradation	[212]
miR-21	GDF-5	Inhibit chondrocyte proliferation	[213]
miR-146a	Smad4	Promote chondrocyte apoptosis	[214]
miR-146b	A2M	Promote chondrocyte apoptosis	[215]
miR-181a	GPD1L	Promote chondrocyte apoptosis	[216]
miR-210	HIF-3α	Promote chondrocyte proliferation, increase ECM deposition	[217]
miR-146	TNF-α	Inhibit inflammatory responses	[218]
miR-9	NF-κB	Inhibit inflammatory responses	[219]
miR-558	COX-2	Inhibit inflammatory responses, inhibit ECM degradation	[220]

## 5. Conclusions

In this review, we first presented evidence that mitochondria not only function as “energy powerhouses”, but also regulate several physiological activities of chondrocytes, including oxidative stress, autophagy, apoptosis, chondrocyte senescence, and calcium homeostasis in OA. In addition, more and more evidence has revealed the connection involving the AMPK–SIRT signaling pathways, mitochondria dysfunction, and OA pathogenesis. The AMPK–SIRT signaling pathways help to regulate mitochondrial bioactivities and chondrocyte viability, including ECM production, mitochondrial biogenesis, autophagy, mitophagy, oxidative stress, and inflammation inhibition, suggesting that the AMPK–SIRT signaling pathways could be potential targets for developing new OA therapeutic strategies. Furthermore, a growing number of findings have shown that lncRNA and miRNA have great potential to be the options for OA treatment because of their involvement in several important pathways related to OA pathogenesis, including the regulation of ECM degradation, chondrocyte apoptosis, chondrocyte proliferation, inflammatory responses, and autophagy. Based on the above findings, we found a lot of drugs that target different signaling pathways. However, we need further research to better understand the downstream mechanisms of the AMPK–SIRT signaling pathways, lncRNA, and miRNA to pursue new pharmacologic targets for OA pathogenesis.
